# Supplementary material for: The Molecular Drivers of Honey Robbing in Apis mellifera L.: Morphological Divergence and Oxidative-Immune Regulation Mechanisms Based on Proteomic Analysis
Source: Insects. 2025 Sep 22;16(9):987. doi: 10.3390/insects16090987 (PMC12471248; doi:10.3390/insects16090987)
Supplement: Supplementary file 1 [file insects-16-00987-s001.zip › Supplementary File S1.pdf]

Table S1. The mean and standard deviation of morphological indicators for control bee and robber bee.

	Control Bee	Robber Bee
Pigmentation of tergite 2	7.50±1.72	5.20±2.44 *
Pigmentation of tergite 3	6.90±1.85	3.90±2.96 *
Pigmentation of tergite 4	2.20±1.32	1.40±1.26
Pigmentation of scutellum, Cupolla	5.40±0.52	5.10± 1.37
Pigmentation of scutellum, B and K	1.65±1.98	1.20±1.74
Pigmentation of labrum 1	0.20±0.42	0.10±0.32
Pigmentation of labrum 2	0.80±0.92	0.50±0.97
Width of tomentum on tergite 4	0.93±0.07	0.83±0.06 **
Width of stripe posterior of tomentum	0.47±0.06	0.51±0.03
Length of cover hair on tergite 5	0.20±0.01	0.20±0.02
Proboscis	6.10±0.52	6.09±0.41
Length of hind leg fermur	2.37±0.10	2.35±0.10
Length of hind leg tibia	2.89±0.23	2.86±0.12
Length of hind leg metatarsus	2.09±0.07	2.04±0.10
Width of hind leg metatarsus	1.10±0.06	1.07±0.08
Length of sternite 6	2.70±0.07	2.74±0.09
Width of sternite 6	2.83±0.12	2.88±0.12
Length of sternite 3	2.84±0.09	2.80±0.21
Wax mirror of sternite 3 longitudinal	1.44±0.03	1.44±0.01
Distance between wax mirrors st. 3	0.25±0.00	0.25±0.00
Wax mirror of sternite 3 transversal	2.14±0.02	2.14±0.01
Fore wing length	9.35±0.14	9.30±0.23
Fore wing width	3.22±0.09	3.17±0.11
Number of hindwing hook	21.30±1.16	20.40±1.51

Note: data represent the mean ± SD, and statistical analyses were performed using the *T*-test, * represents $P < 0.05$, and ** represents $P < 0.01$.
